# Probationer Nurses

**Published:** 1890-07-19

**Authors:** 


### Passing Topics.

### PROBATIONER NURSES.

It was to be expected that an inquiry like that now proceeding before the Committee of the House of Lords, and which, we trust, may prove as useful as it bids fair to be exhaustive, would bring to the surface everybody with a grievance, and we shall scarcely err if we place in that category the nurses who have recently figured so prominently. We do not venture to offer an opinion as to whether their complaints are well founded. It might amount to a contempt of court if we did. The questions raised concerning which there is a conflict of evidence may well be left to the Committee, who, it is only just to say, appear determined, no matter how tedious the process, to arrive at the truth.

But there is one portion of the subject where the facts are not in dispute. We allude to the employment of probationer nurses, and especially of paying probationers, in the wards of our hospitals. We have no particular hospital in view; we believe all are more or less inclined to admit both classes, and we fear that at all alike there may be a little tendency to rely too much upon them. Two causes contribute to this: (1) A desire for economy; ordinary probationers receive but little wages during the first year, while those who pay besides receiving no wages may be said to provide their own board; and (2) the real or supposed exigencies of the self-imposed task of training nurses, which occasionally means keeping up the supply for the nursing institution attached to the hospital and "run" as a source of revenue.

The training of nurses is undoubtedly a valuable function. Nursing is an art which cannot be self taught or taught without subjects. The old notion that every woman is a born nurse has been altogether exploded. A long course of arduous training is required before efficiency is attained, and the hospitals alone supply the necessary opportunities. Yet the hospitals have been no more established in order to train nurses than to educate medical students. They exist primarily as philanthropic institutions for the relief and cure of the patients admitted to their benefits. Teaching and training are but secondary duties. The question, therefore, immediately arises whether it is to the interest of the patients that there should be a constant passage of nurses through the intermediate stages of training. Those with practical knowledge will scarcely hesitate as to the answer. Too frequent change, even when one efficient supplants another, is in the nature of an evil; the constant substitution of untrained for trained nurses is a process of dilution which is apt to reduce efficiency much below proof.

The hospitals are not altogether to blame. The introduction into nursing of a class of women superior to that known to a previous generation has brought with its many advantages certain drawbacks. Among others it has opened the door to a restless, self-conscious, and ambitious element out of place in a calling which, for its highest fulfilment, demands a large measure of personal suppression and self-sacrifice. Nurses are not always content with a sense of duty performed, while some of them are independent of their salaries. Many pine for new experiences, and regard continuous service in one institution as dull and profitless. Frequent changes appear, therefore, inevitable, but the hospitals need not aggravate their difficulties by helping to perpetuate the conditions which foster them. Miss Lückes says that fifteen hundred applications for probationerships were received at the London during a single year. We have heard of a still larger number at another hospital. It will be a happy thing for nursing when the "craze" which has rendered it fashionable dies out, and we have nothing but reproof for the women who take to it with any but a serious intent. Still, we cannot help seeing that with this enormous body of applicants to choose from, it is the hospitals' own fault if they fail to secure the right kind of material. There ought to be a distinct rule as to the maximum proportion the probationers of less than a year's standing should bear to the rest of the staff. As to the paying probationers, if we consider nothing more than the patients' comfort and well-being, we should say the hospitals would be better without them. No doubt there are instances, which should be provided for, in which knowledge of nursing is legitimately sought by those who cannot remain a term of two or three years, but it is neither desirable nor even seemly to admit to hospital work every woman who is willing to pay a fee of 13 guineas for three months' board and lodging. There are the gravest objections, moral and material, to be urged against too great liberty in this matter. It is impossible to be too careful to exclude any but strictly suitable women of sufficient age, and if right is to be done alike to the patients and medical officers, this class of probationers ought never to be anything but supernumary to the regular nursing staff.

